# Direct oral anticoagulants in pediatric venous thromboembolism: Review of approved products rivaroxaban and dabigatran

**DOI:** 10.3389/fped.2022.1005098

**Published:** 2022-10-13

**Authors:** Maha Al-Ghafry, Anjali Sharathkumar

**Affiliations:** ^1^Division of Pediatric Hematology and Oncology, Royal Hospital, Muscat, Oman; ^2^Division of Pediatric Hematology and Oncology Stead Family Department of Pediatrics, University of Iowa Carver College of Medicine, Iowa City, IA, United States

**Keywords:** direct oral anticoagulants (DOACs), venous thromboembolism (VTE), rivaroxaban, dabigatran, thrombosis

## Abstract

Venous thromboembolism is a major hospital acquired complication in the pediatric population over the last two-decades, with a 130% increase in the past decade. Direct oral anticoagulants (DOACs) are a newer class of anticoagulant medication for the treatment and prophylaxis of VTEs that provide the primary advantages of an oral route of administration without a requirement to adjust dosing to achieve a therapeutic level. It is anticipated that these medications will quickly replace parenteral anticoagulants and clinicians should familiarize themselves with DOACs. In this article, we provide an overview of the pharmacological properties of DOACs, with a specific focus on rivaroxaban and dabigatran, which have been approved for use in pediatric patients. Each drug's characteristics are discussed along with data from their respective clinical trials.

## Introduction

Venous thromboembolism (VTE) has emerged as a major hospital acquired complication in the pediatric population over the last two-decades. This is primarily related to advances in supportive interventions leading to disruption of Virchow's triad. Estimates show a 70% increase in the rate of hospitalized children diagnosed with VTE between 2001 and 2007 (from 18 per 100,000 hospitalized children in 1990s to 50 in 2000s) (1), and a further 130% increase from 2008 to 2019 (46 VTE cases per 10 000 hospitalizations to 106 in 2019) (2). A dedicated pediatric hemostasis-thrombosis inpatient consult service has noted, between 2011 and 2020, a 7-fold increase in daily census and 10-fold increase in consults for thromboprophylaxis, with the highest percentage of consult requests from orthopedic surgery and the cardiovascular intensive care unit (3). Therefore, anticoagulation therapy has become critical for management of these children, whether for the primary prevention or treatment of VTE.

Available therapeutic agents for the management of pediatric VTEs consist of a single oral medication (vitamin K antagonist) with the remainder being parenteral preparations: intravenous (IV) unfractionated heparin (UFH), subcutaneous (SQ) low-molecular weight heparin (LMWH), SQ fondaparinux, and IV direct thrombin inhibitors (DTIs) such as bivalirudin and argatroban (4); LMWH is the most commonly used medication for inpatient and outpatient VTE management. These medications require titration to therapeutic levels, multiple blood draws (some with strict time-based level collections) and subsequent medication dose adjustments to reach the desired levels. This not only worsens children's trypanophobia but can increase hospital length of stay and delay discharges.

Direct oral anticoagulants (DOACs) are a newer class of anticoagulant medication for the treatment and prophylaxis of VTEs that provide the primary advantages of an oral route of administration without a requirement to adjust dosing to achieve a therapeutic level. It is anticipated that these medications will quickly replace parenteral anticoagulants, specifically LMWH, and clinicians should familiarize themselves with DOACs. In this article, we provide an overview of pharmacological properties of DOACs with a specific focus on two DOACs, rivaroxaban and dabigatran, which have been approved for use in pediatric patients with VTE. Both DOACs have been approved by the Food and Drug Administration (FDA) in the United States and by the European Medicines Agency (EMA) in the European Union.

## Development of oral anticoagulants and mechanism of action of DOACs

Anticoagulants were first used in the parenteral form in the 1940s with the discovery of UFH, with LMWH not in use until the 1980s, and the next advancement being in the 1990s with IV DTIs (bivalirudin and argatroban) (5). The first oral anticoagulant, warfarin, was initially licensed in 1954, and it gained popularity in patients with non-valvular atrial fibrillation for prevention of embolic strokes (6). Its use has been precarious due to the difficulty in maintaining therapeutic levels, with the need for frequent monitoring, along with complications of major and fatal bleeding events due to supratherapeutic dosing. It was another 70 years before the second oral anticoagulant (dabigatran) was approved in 2010. Since then, DOACs have revolutionized VTE treatment and thromboprophylaxis in many adult patient populations [currently contraindicated in patients with mechanical heart valves and during pregnancy] (7). Currently approved DOACs in adults are dabigatran etexilate (2010), rivaroxaban (2011), apixaban (2012), edoxaban (2014) and betrixaban (2017) (8).

DOACs exert their anticoagulant properties by affecting the common pathway in the coagulation cascade, with the two major oral medication classes being direct factor Xa inhibitors (DFXaI) and direct thrombin inhibitors (DTIs). An advantage of DOACs is their selective binding to their target factors (FXa for DFXaI and thrombin for DTIs) without the need for a cofactor. Conversely, medications such as indirect FXa inhibitors (e.g., heparins or fondaparinux), exert their anticoagulant effect by potentiating the cofactor antithrombin to bind to FXa (along with thrombin for UFH), thereby neutralizing their procoagulant effects by being removed from circulation (9).

**Figure 1:**
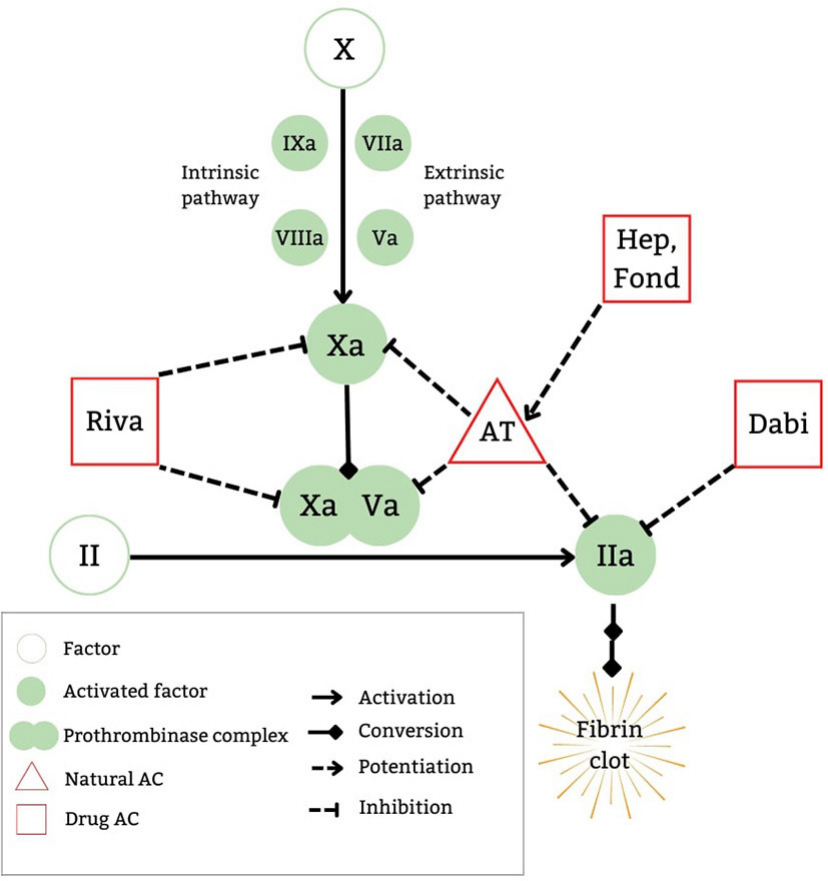
DOAC mechanism of action. AC, anticoagulatnt; AT, antithrombin; Dabi, dabigatran etexilate; Fond, fondaparinux; Hep, heparins; Riva, rivaroxaban.

Within the common pathway (Figure 1), FX is activated to FXa which in turn cleaves prothrombin to thrombin, whereby 1 molecule of FXa generates 1,000 molecules of thrombin. The prothrombinase complex (FXa-FVa complex) accelerates this activation 300,000 fold (10). This thrombin generation in turn re-potentiates the prothrombotic pathway by accelerating further activation of the cascade with continued thrombin generation. Therefore, direct inhibition of FXa or thrombin ultimately leads to significant reduction in thrombin generation and the resultant fibrin clot formation.

Rivaroxaban and dabigatran etexilate are currently the first two DOACs approved for use in pediatric patients with VTE by the FDA and EMA. Pediatric dosing regimens are generally extrapolated from adult trial data; however, legislative changes have increased pediatric-specific data. The Best Pharmaceuticals for Children Act and Pediatric Research Equity Act have pushed to assess dosing and adverse events in pediatric patients via their own clinical trials (11), with rivaroxaban and dabigatran etexilate being amongst the first drugs to benefit from their own pediatric trials.

Below is a comparison of these two DOACs with their relevant trial data.

## Rivaroxaban

Rivaroxaban is a small molecule that reversibly binds directly to the S1 and S4 sites on FXa (12), with its advantage over indirect FXa inhibitors being its ability to bind both free FXa and FXa bound within the prothrombinase complex, thereby exerting a greater anticoagulant effect. It was initially EMA and FDA approved for adults in 2011, with pediatric approvals by EMA in 2020 (13) and the FDA in 2021 (14). The drug characteristics, pharmacokinetics, pharmacodynamics, safety and efficacy were studied to assess its safety and establish recommended dosage. Patients included were those up to 18 years of age with confirmed VTE—Table 1 (15–21). Neonates included were those at least 37 weeks of gestational age and who have enterally fed for at least 10 days, with a minimum weight of 2.6 kg. This group received a newly formulated oral suspension, in addition to the previously available tablets. Children with active malignancy and those on non-steroidal anti-inflammatory or antiplatelet agents were also eligible (21). The studies excluded patients with active/risk of bleeding, liver and/or renal dysfunction, those pregnant or breastfeeding, patients on strong inhibitors of both CYP3A4 and P-glycoproteins, and those using strong inducers of CYP3A4 (complete and detailed list of contraindications and patient exclusions is in Table 2) (16, 22).

**Table 1:** DOAC characteristics, pharmacodynamics, and pharmacokinetics.

	**Rivaroxaban**	**Dabigatran etexilate**
Trade name (pharmaceutical company)	Xarelto (Janssen Pharmaceuticals)	Pradaxa (Boehringer Ingelheim)
Category	Direct factor Xa inhibitor	Direct thrombin inhibitor
EMA approval	2020	2020
FDA approval	2021	2021
Minimum age	Birth (at least 37 weeks gestational age and 10 days of enteral feeds)	3 months (as soon as able to swallow soft foods)
Minimum weight	2.6 kg	3 kg
Dosing	*Weight based dosing (see Table 3)*	*Age and weight-based dosing (see Table 5)*
Formulations	- Oral solution: 1 mg/mL - Tablets: 2.5 mg, 10 mg, 15 mg, 20 mg	Dosage forms cannot be substituted for each other - Capsules: 75 mg, 110 mg, 150 mg - Oral pellets: 20 mg, 30 mg, 40 mg, 50 mg, 110 mg, 150 mg - Oral solution: not commercially available
Route	Oral	Oral
Drug form	Active drug	Produrg
Bioavailability	80%	7% (bioavailability varies between dosage forms, but is generally very low)
Half-life	7–11 hours	9–11 hours
Excretion	Renal (66%), Fecal (28%)	Fecal (85%)
Elimination	Urine (36%), fecal (7%), hydrolytic cleavage (14%), oxidative pathways (32%)	Renal (80%)
Drug interactions	Dual P-glycoprotein & CYP3A4 inducers and inhibitors	P-glycoprotein inducers and inhibitors
Monitoring	Not needed	Not needed
Coagulation test effect	Prolongation of PT and aPTT	Prolongation of PT and aPTT

AC, anticoagulation; ALT, alanine aminotransferase; AP, alkaline phosphatase; aPTT, activated partial thromboplastin time; AST, aspartate aminotransferase; Cr, creatinine; eGFR, estimated glomerular filtration rate; GI, gastrointestinal; Hb, hemoglobin; Plt, platelet; PT, prothrombin time; ULN, upper limit of normal.

**Table 2:** DOAC contraindications, administration, and adverse effects.

	**Rivaroxaban**	**Dabigatran etexilate**
Contraindications *(studies did not include patients with the following)*	- Active bleeding/high risk of bleeding - eGFR <30 mL/min/1.73 m^2^ (<1 year of age) - Cr >97.5th %ile - Liver dysfunction with coagulopathy *or* ALT > 5× ULN *or* total bilirubin >2× ULN with direct >20% - Use with dual P-glycoprotein/CYP3A4 inhibitors, e.g., azole-antimycotics or HIV protease inhibitors - Use with CYP3A4 inducers (e.g., rifampicin, phenytoin) - Those of childbearing potential without proper contraception, pregnancy or breastfeeding	- Active bleeding/high risk of bleeding - eGFR <50 mL/min/1.73 m^2^ (12–18 years) *or* eGFR <80 mL/min/1.73 m^2^ (0–12 years) *or* dialysis - Liver dysfunction with active hepatitis A, B or C *or* ALT, AST or AP > 3 × ULN - Prosthetic heart valve (that requires AC) - Active infective endocarditis - Hb <8 g/dL or Plt <80 × 10^9^/L - Use with P-glycoprotein inducers (e.g., rifampin) - Use with P-glycoprotein inhibitors (e.g., azole-antimycotics or HIV protease inhibitors) not studied
Renal impairment contraindication	Yes	Yes
Hepatic impairment contraindication	Yes	No
Administration	- With food	- With/without food - Mix only with apple juice, baby rice cereal (prepared with water), mashed carrots or potatoes - Do not administer with milk products - Do not administer via a syringe or feeding tube
Formulations	Suspension and capsules	Pellets and tablets
Adverse effects, common	- Bleeding: GI tract, oral cavity, nasal, genital, skin - Anemia - Abdominal pain, diarrhea, nausea, emesis - Nasopharyngitis, cough, chest pain - Extremity pain - Headaches - Menorrhagia, epistaxis - Pyrexia, fatigue	- Bleeding: GI tract, oral cavity, procedural site, injection site - Anemia - Abdominal pain (upper), emesis, dyspepsia - Nasopharyngitis, respiratory tract infection - Extremity pain - Headaches - Epistaxis - Pyrexia
Peri-procedural discontinuation	- Low risk of bleeding: 24 h prior - High risk of bleeding: 48 h prior	- Low risk of bleeding: 24 h prior - High risk of bleeding: 48 h prior
Reversal agent	- Andexanet alfa *(no studies in pediatric patients)*- PCCs can be considered	Idarucizumab *(under investigation for pediatric patients)*

AC, anticoagulation; ALT, alanine aminotransferase; AP, alkaline phosphatase; aPTT, activated partial thromboplastin time; AST, aspartate aminotransferase; Cr, creatinine; eGFR, estimated glomerular filtration rate; GI, gastrointestinal; Hb, hemoglobin; PCC, prothrombin complex concentrate; Plt, platelet; PT, prothrombin time; ULN, upper limit of normal.

Table 1 also lists rivaroxaban's pharmacodynamics and pharmacokinetics: it has 80% bioavailability (22), reaching its peak plasma concentration within 2–4 h, with a half-life of 7–11 h (12). Its excretion is predominantly renal (66%) with the remainder fecal (28%), with elimination being multifactorial: 36% renal, 7% fecal, 14% via hydrolytic cleavage and 32% via oxidative pathways (23). Rivaroxaban is to be taken with meals (due to studies that showed decreased bioavailability at higher doses) (22), and most of those surveyed on the suspension found it to be palatable (16). There is no requirement for direct or indirect monitoring of therapeutic levels, although there is prolongation of both prothrombin time (PT) and activated partial thromboplastin time (aPTT) (12): Anti-Xa levels can be measured in some high risk patients if there is concern about the pharmacodynamics of the drug (24). Drug dosing is noted in Table 3.

**Table 3:** Rivaroxaban dosing [modified from Young et al. (16) and Rivaroxaban package insert].

**Dosing**		
**Treatment dosing (after completion of 5 days of parenteral treatment)**	**Weight (kg)**	**Dose (mg)**
	2.6–2.9	0.8 TID
	3–3.9	0.9 TID
	4–4.9	1.4 TID
	5–6.9	1.6 TID
	7–7.9	1.8 TID
	8–8.9	2.4 TID
	9–9.9	2.8 TID
	10–11.9	3 TID
	12–29.9	5 BID
	30–49.9	15 OD
	≥50	20 OD
**Thromboprophylaxis dosing**	**Weight (kg)**	**Dose (mg)**
	7–7.9	1.1 BID
	8–9.9	1.6 BID
	10–11.9	1.7 BID
	12–19.9	2 BID
	20–29.9	2.5 BID
	30–49.9	7.5 OD
	≥50	10 OD

BID, twice a day; OD, once a day; TID, three times a day.

Comparing rivaroxaban to standard of care (SOC) in the EINSTEIN-Jr phase 3 trial, Male et al. (15) found that rivaroxaban had similar efficacy to that of SOC—Table 4. In this 2:1 randomized open label trial, 500 pediatric patients were enrolled between November 2014 and September 2018 in 107 institutions across 28 countries; the greatest proportion of patients were in the 12 to <18 years of age category (~55%). Patients studied included those with active malignancy, major organ disease, cardiac, gastrointestinal, renal and neurologic risk factors, known thrombophilia (inherited or acquired), and those on estrogens or progestins (15). Patients received 5–9 days of parenteral therapy with SOC (UFH, LMWH or fondaparinux) prior to being randomized, and they were then followed for the duration of their treatment course of 3 months (or 1 month for those <2 years of age with catheter related thrombosis). Six patients did not initiate study medication and 7 patients did not continue in the trial after initiation in the rivaroxaban group whereas 3 patients did not initiate study medication and 6 patients did not continue in the trial after initiation in the SOC group.

**Table 4:** Rivaroxaban and dabigatran etexilate trial comparison.

	**Einstein Jr phase 3**,		**DIVERSITY phase 3**,	
	***N*** = **500**		***N*** = **267**	
	**(** **15** **)**		**(** **25** **)**	
DOAC	Rivaroxaban		Dabigatran etexilate	
Institutions and Countries	107 institutions in 28 countries		65 institutions in 26 countries	
Enrollment period	2014, Nov to 2018, Sept		2014, Feb to 2019, Nov	
Prior parenteral therapy duration	5–9 days of UFH, LMWH or fondaparinux		5–21 days of UFH or LMWH	
Study treatment period	3 months (1 month for <2 years of age with catheter related thrombosis)		3 months	
Patients enrolled	500		267	
Randomization (intervention:SOC)	2:1		2:1	
Trial type	Open label		Open label	
	**Patients, N**			
	**Intervention**	**SOC**	**Intervention**	**SOC**
Patients randomized, included in efficacy analysis	335	165	177	90
Parenteral therapy duration mean prior to randomization, days	*Not defined*		15.7	14.8
Anticoagulation received	Rivaroxaban	UFH, LMWH, fondaparinux, or VKA	Dabigatran	UFH, LMWH, fondaparinux or VKA
**VTE site (%)**				
Cerebral venous or sinus thrombosis	74 (22%)	43 (26%)	20 (11%)	6 (7%)
Catheter related	90 (27%)	37 (22%)	27 (15%)	20 (22%)
Non-catheter related	171 (51%)	85 (52%)	110 (62%)	60 (67%)
Pulmonary embolism	*Not defined*		20 (11%)	4 (4%)
**Age distribution, years (%)**				
0 to <2	37 (11%)	17 (10%)	22 (12%)	13 (14%)
2 to <12	114 (34%)	56 (34%)	43 (24%)	21 (23%)
12 to <18	184 (55%)	92 (56%)	112 (63%)	56 (62%)
Median exposure, days	91 days (3-month treatment) and 31 days (1-month treatment)		84.5 days	85 days
Patients included in safety analysis^@^	329	162	176	90
**Adverse events (%)**				
Bleeding, major^*^	0 (0%)	2 (1%)	4 (2%)	2 (2%)
Bleeding, CRNM	10 (3%)	1 (<1%)	2 (1%)	1 (1%)
Treatment related deaths	0 (0%)	0 (0%)	0 (0%)	0 (0%)
**Thrombus endpoint^#^ (%)**				
Thrombus resolution, complete	128 (38%)	43 (26%)	81 (46%)	38 (42%)
Thrombus, recurrence	4 (1%)	5 (3%)	7 (4%)	7 (8%)

@ Einstein Jr intervention, 6 patients did not initiate study medication and 7 patients did not continue in the trial after initiation; Einstein Jr SOC, 3 patients did not initiate study medication and 6 patients did not continue in the trial after initiation; Diversity intervention, 1 patient did not initiate study medication and 8 patients did not continue in the trial after initiation; Diversity SOC, 5 patients did not continue in the trial after initiation.

* Major bleeding defined by Halton et al. (25) as fatal bleeding or ≥2 g/dL drop in hemoglobin in 24 h.

# Remainder of thrombi were undefined.

CRNM, clinically relevant non-major; LMWH, low molecular weight heparin; SOC, standard of care; UFH, unfractionated heparin; VKA, vitamin K antagonist.

No patients in the rivaroxaban group had a major bleeding event while there were 2 (1%) in the SOC group (one intracranial and the other pulmonary), with 3% and <1% of patients having clinically relevant non-major bleeding (CRNMB) events in the intervention compared to SOC groups, respectively. Non-bleeding adverse events were mostly mild-moderate (summary of common adverse events in Table 2). Thrombus resolution occurred in 128 (38%) patients in the rivaroxaban group and 43 (26%) in the SOC group, which was clinically significant (*p* < 0.05), with a recurrence rate of 1 and 3%, respectively. There were no treatment related deaths.

Based on the pharmacokinetic data, peri-procedurally, rivaroxaban should be discontinued 24 and 48 h prior to procedures with low and high bleeding risks, respectively (26). For acute and emergent reversal of rivaroxaban in cases with life-threatening bleeding, andexanet alfa has been approved in adult patients in 2018. Andexanet alfa is an inactive recombinant factor Xa, and in the ANNEX-4 study (27), 80% of patients with acute major bleeding (intracranial or gastrointestinal) secondary to an oral DFXaI, achieved improved hemostatic function with administration of andexanet alfa. The reversal agent is expensive and not readily available in many institutions, with prothrombin complex concentrates (PCCs) being an alternative therapy (28). As there are no current pediatric specific data on its use, a recent survey of pediatric hematologists found a 44% preference for the use of andexanet alfa in pediatric patients requiring reversal for acute life threatening bleed due to FXaI, with 55% choosing PCCs (29). A newer investigational drug, ciraparantag, can reversibly bind and neutralize the effects of DFXaI, DTIs and heparins (30), with initial phase 2 clinical trials showing promise (31).

## Dabigatran etexilate

Dabigatran etexilate, a DTI, is a prodrug which is converted to its active form dabigatran by carboxylesterases (32). It reversibly binds directly to the S1 site on thrombin (12), thereby inhibiting it's further role in clot formation and the positive feedback in propagating factors. It was the first DOAC to be approved by the FDA (in 2010) and EMA (2011), and was subsequently the first DOAC to be approved in pediatric patients by both EMA in 2020 (33) and the FDA in 2021 (34). The drug characteristics, pharmacokinetics and pharmacodynamics were well-studied in pediatric patients to assess its safety and establish dosage recommendations—Table 1 (25, 35–38). Its use was assessed in patients 3 months up to 18 years of age with confirmed VTE using an oral suspension, pellets or capsules (25). Patients excluded from the study (25) were those with active/risk of bleeding, liver and/or renal dysfunction, those with a prosthetic heart valve that requires AC, those with active infective endocarditis, patients who are anemic and/or thrombocytopenic, and avoided in those on P-glycoprotein inducers or on P-glycoprotein inhibitors (39) (complete and detailed list of contraindications is in Table 2).

Dabigatran etexilate has low bioavailability of 7% per initial studies (40), although there are differences in bioavailability of the different dosage forms, therefore they cannot be substituted for each other (39). It has a peak effect of 1–2 h (delayed peak with meals high in fat content) with a half-life of 9–11 h (for pellets), and is excreted mainly unchanged in the feces (86%), with the active dabigatran primarily eliminated in the urine (80%) (40). Capsules and oral pellets are currently available for use (the oral solution is not commercially available). Oral pellets (39) can be administered in children as early as those who are able to swallow soft, solid foods by mixing with only the following: apple juice, baby rice cereal (prepared with water) and mashed carrots or potatoes. The pellets should not be administered with any milk products, should not be administered via a syringe or feeding tube, and should be administered within 30 minutes of mixing with juice or food. There is no requirement for direct or indirect monitoring of therapeutic levels due to predictable pharmacokinetics and pharmacodynamics (12), although there is prolongation of both PT, aPTT and thrombin time (TT) (41). Drug dosing is noted in Table 5.

**Table 5:** Dabigatran etexilate dosing (modified from dabigatran etexilate package insert).

**Dosing**				
**Treatment dosing (after completion of 5 days of parenteral treatment**	**Age**	**Weight (kg)**	**Age (months)**	**Dose (mg)**
	**<2 years**	3–3.9	3–5.9	30 BID
		4–4.9	3–9.9	40 BID
		5–6.9	3–4.9	40 BID
			5–23.9	50 BID
		7–8.9	3–3.9	50 BID
			4–8.9	60 BID
			9–23.9	70 BID
		9–10.9	5–5.9	60 BID
			6–10.9	80 BID
			11–23.9	90 BID
		11–12.9	8–17.9	100 BID
			18–23.9	110 BID
		13–15.9	10–10.9	100 BID
			11–23.9	140 BID
		16–20.9	12–23.9	140 BID
		21–25.9	18–23.9	180 BID
	**Age**	**Weight (kg)**		**Dose (mg)**
	**2–11.9 years**	7–8.9		70 BID
		9–10.9		90 BID
		11–12.9		110 BID
		13–15.9		140 BID
		16–20.9		170 BID
		21–40.9		220 BID
		≥41		260 BID
Thromboprophylaxis dosing	Not available			

BID, twice a day.

The pediatric dabigatran trial (DIVERSITY) assessed dabigatran safety (37), with Halton et al. (25) showing the non-inferiority of dabigatran to SOC for the secondary prevention of VTE. The open label trial—Table 4—assessed 267 patients in 65 institutions across 26 countries from February 2014 to November 2019. Patients received 5–21 days of parenteral therapy with UFH, LMWH, fondaparinux or VKA (mean of ~15 days) prior to randomization and followed for 3 months (treatment duration). Patients aged 3 months to 18 years of age (the majority of patients were in the 12 to <18 years age group at 62%) were randomized 2:1 with 177 receiving dabigatran and 90 receiving SOC. Patients in the study included those with previous thromboembolism, thrombophilia (inherited or acquired) and those with congenital heart disease, malignancy, heart failure and diabetes. In the dabigatran group, 1 patient did not initiate the study medication and 8 patients did not continue in the trial after initiation, while in the SOC group, 5 patients did not continue in the trial after initiation.

Major bleeding (2%) and CRNMB (1%) events were the same in both groups; major bleeding events were procedural, urogenital, GI, and intracranial in the dabigatran group and retroperitoneal and GI in the SOC group. The common, non-bleeding adverse events are noted in Table 2. Thrombus resolution was achieved in 46% of those receiving dabigatran and 42% of those on SOC, with 4% and 8% having thrombus recurrence, respectively. There was 1 on-treatment death that was not related to treatment in the SOC group.

Based on pharmacokinetic data, peri-procedurally, dabigatran should be discontinued 24 and 48 h prior to procedures with low and high bleeding risks, respectively (42), in those with appropriate renal function. For acute and emergent reversal in cases of severe hemorrhage, idarucizumab, a monoclonal antibody that binds to dabigatran, has shown promise. In the RE-VERSE AD trial (43), emergent use of idarucizumab in patients with uncontrolled bleeding (or those who required urgent procedural intervention) was found to provide safe and durable reversal. Idarucizumabis is currently being investigated for its use as a reversal agent in children (44).

## Conclusion

It is evident that DOACs administered to pediatric patients have provided similar safety profiles and efficacy in VTE management compared to SOC with traditional anticoagulants such as heparins and VKAs. The EINSTEIN-Jr and DIVERSITY trials have been instrumental in not only establishing the safety and efficacy of rivaroxaban and dabigatran, respectively, in pediatric patients, but in furthering the call for pediatric-specific interventional studies for newer therapeutics in pediatric hematology and beyond. Additional data on treatment and thromboprophylaxis in special patient populations at high risk for thrombosis is warranted. This is particularly for pediatric patients with heart disease (congenital and acquired) due to the multiple procedures they undergo and the use of prosthetic heart valves, which limits their potential use of DOACs. The trials also likely inadvertently excluded patients with active malignancy due to their repeat anemia and thrombocytopenia. Whitworth and Raffini provide further clinical aspects to be considered in these special patient populations (45).

Other DOACs (Apixaban, Betrixaban, Edoxaban) are currently under investigation in pediatric patients (46).

With the introduction of DOACs, the oral route of administration without the need for therapeutic monitoring is a triumph for patients and their healthcare teams alike. This data, along with newer studies such as the Kids-DOTT trial (showing the non-inferiority of 6 weeks vs. 3 months of AC in pediatric patients with provoked VTE) (47), is heralding an optimistic era in treating pediatric VTE.

## Author contributions

MA-G and AS contributed to the conception and design of the manuscript. MA-G completed the initial literature review and wrote the first draft of the manuscript. Both authors contributed to the manuscript revision. Both authors contributed to the article and approved the submitted version.

## Conflict of interest

The authors declare that the research was conducted in the absence of any commercial or financial relationships that could be construed as a potential conflict of interest.

## Publisher's note

All claims expressed in this article are solely those of the authors and do not necessarily represent those of their affiliated organizations, or those of the publisher, the editors and the reviewers. Any product that may be evaluated in this article, or claim that may be made by its manufacturer, is not guaranteed or endorsed by the publisher.

## Abbreviations

AC: anticoagulation
CRNMB: clinically relevant non-major bleeding
DOAC: direct oral anticoagulant
DFXaI: direct factor Xa inhibitor
DTI: direct thrombin inhibitor
IV: intravenous
LMWH: low molecular weight heparin
SQ: subcutaneous
SOC: standard of care
UFH: unfractionated heparin
VTE: venous thromboembolism.
